# Community-Based, Rapid HIV Screening and Pre-Exposure Prophylaxis Initiation: Findings From a Pilot Program

**DOI:** 10.7759/cureus.20877

**Published:** 2022-01-02

**Authors:** David H Schaffer, Lindsey M Sawczuk, Hui Zheng, Wendy L Macias-Konstantopoulos

**Affiliations:** 1 Department of Emergency Medicine, Massachusetts General Hospital, Boston, USA; 2 Department of Medicine, Tulane University School of Medicine, New Orleans, USA; 3 Department of Emergency Medicine, Harvard Medical School, Boston, USA

**Keywords:** pre-exposure prophylaxis (prep), community-based, linkage to care, community health research, hiv testing

## Abstract

Objective

Many individuals do not have regular access to medical care and preventative health services, suggesting the need for alternative access to HIV testing and pre-exposure prophylaxis (PrEP). The purpose of this study is to describe a novel, community-based HIV screening, a PrEP initiation program, and report preliminary findings.

Methods

One Tent Health, a 501(c)(3) nonprofit organization, launched a pop-up HIV screening and PrEP initiation program in high-risk areas of Washington, DC in 2017. We describe the unique features of the program and report 25 months of screening, risk assessment, and PrEP education data. Odds ratios were calculated to identify disparities in both HIV risk factors and prior HIV testing.

Results

Between October 2017 and November 2019, 846 individuals underwent HIV screening. Six individuals (0.709%) screened HIV-positive. Approximately 13% had never been screened for HIV, and another 13% had at least one major risk factor for HIV. Individuals who self-identified as White were more likely to have risk factors (OR 2.19, p = 0.0170) and less likely to have ever been tested (OR 0.50, p = 0.0409). Individuals who self-identified as Black or African American were less likely to have risk factors for HIV (OR 0.57, p = 0.0178). Disparities by sex and gender were also observed.

Conclusions

This program appears to be the first of its kind within the United States. We found the program to be cost-effective, well-received by the community, and accessible by high-risk and unreached populations while further revealing the role of race and gender in the HIV epidemic.

## Introduction

Early diagnosis and treatment remain key components of reducing transmission and improving the health of people living with HIV (PLWH) [1]. In the United States (US), ongoing localized epidemics require innovative disease burden reduction strategies. Washington, DC has the highest incidence and prevalence rates of HIV infection as compared to all states in the US [2]. Prevalence estimates indicate approximately 2% - 3% of the DC population are PLWH, potentially double or triple the WHO threshold for epidemic status [2-4]. Further, the incidence of new HIV cases in DC did not decline between 2014 - 2018 [4].

The history of this localized epidemic is complex, though it is clear that HIV rates correlate with factors such as income, racial background, and sexual partners [5-6]. Governmental programs focused on risk reduction have demonstrated uptake; for example, the DC Department of Health reported that in 2016 over five million male and female condoms were distributed, while nearly one million used needles were collected [7]. Yet, gaps in HIV prevention persist; nearly 25% of those who screened HIV-positive remained without linkage to care (LTC) within three months of screening [5]. Further, the lifetime risk of acquiring HIV remains as high as one in 13 in DC compared to about one in 100 for the nation as a whole [8]. Ideally, all residents aged 15 to 65 would receive their recommended HIV screening once and subsequent tests as dictated by lifestyle risk factors [9].

Many individuals at high risk for HIV infection do not have regular access to medical care and preventative health services, thus suggesting a need for expanded testing programs [10]. Community-based screening programs in a wide range of settings have previously been explored as a means to address this problem [11-14]. These programs, including mobile, door-to-door, school-based, and other non-facility-based screening, have led to earlier diagnosis, higher average CD4 count at the time of diagnosis, and a higher intake of patients never screened before [13]. In urban settings in the US, those at high risk for HIV were more likely to seek care in these non-traditional screening options [15]. Community-based programs also reported equivalent LTC parameters as compared to facility-based centers [13]. These findings demonstrate the utility of community-based screening options, particularly in high-risk areas.

While community-based programs have many significant advantages, they may also be difficult to maintain. In Cape Town, South Africa, mobile HIV screening was found to be particularly cost-effective, though, ultimately, total operating costs neared $1 million (USD) over a two-year period, which may not be feasible for many healthcare organizations [16]. Implementation of a mobile unit for HIV screening may also be susceptible to other obstacles beyond financial costs, such as licensing, insurance, staff training, and physical space for both parking and screening sessions. 

The present analysis introduces a new screening program designed to minimize the barriers to community-based screening while reaching high-risk populations. This rapid, pop-up HIV screening and pre-exposure prophylaxis (PrEP) initiation program in Washington, DC was launched by One Tent Health (OTH), a 501(c)(3) nonprofit, and began screenings in October 2017. We describe the program below and present an analysis of preliminary data. This pop-up screening model was implemented with the following intentions:

1. Provide free, fast, HIV screening and LTC, regardless of insurance status 

2. Educate clients about PrEP and provide rapid prescriptions when eligible

3. Minimize the socioeconomic, temporospatial, and health literacy barriers that reduce access to HIV screening and PrEP prescription

4. Reduce the stigma surrounding HIV, HIV screening, and PrEP

## Materials and methods

OTH enrolled over 1,600 volunteers predominantly from universities in the Washington, DC area. No healthcare professionals were deployed as sessions included only screening tests waived under the Clinical Laboratory Improvement Amendments of 1988 (CLIA). Areas with high HIV prevalence but without established HIV screening clinics were selected as screening sites. Given the significant burdens of using a mobile van for testing, OTH raised a 10’ x 10’ canvas tent in high foot-traffic areas within these disproportionately affected neighborhoods, typically outside of grocery stores, pharmacies, or laundromats. Five trained volunteers conducted CLIA-waived HIV screening with the INSTI® HIV-1/HIV-2 Rapid Antibody Kit (bioLytical®, Richmond, BC, Canada), a fingerstick kit that shows results in approximately 60 seconds. Volunteers underwent training designed to prepare them with the knowledge and skills necessary to conduct PrEP and HIV education, assess risk factors, discuss results, and a link to care. Volunteers were assigned distinct roles to allow for leadership, outreach, intake, screening, and education.

Upon intake, clients completed a tablet-facilitated demographics questionnaire and assessment of risk factors, prior HIV testing, and knowledge or use of PrEP. Submission of this form electronically relayed the information to the screening volunteer inside the screening tent. PrEP education was conducted and the client then entered the tent and underwent screening. Post-screening disposition was determined by screening results and indications for PrEP initiation. The screening process took approximately 10 - 15 minutes from registration to disposition. LTC was coordinated with clinical partners off-site. When PrEP was indicated, clients who screened negative were able to meet with a clinical partner on-site to review medical history and risk factors and undergo rapid initiation of PrEP. Clients were asked to complete post-screening surveys regarding their experience. 

Clients who screened positive were informed of their results, the need for confirmatory testing, and the importance of initiating care. OTH prioritized same-day confirmatory testing via clinical partners in Washington, DC since same-day confirmation has been associated with accelerated LTC [17]. Clients who declined immediate referrals were offered other options for LTC. All positive screening results were reported to the DC Department of Health to facilitate further outreach and disease surveillance. Follow-up for those clients who screened positive were conducted within one week of screening and again at approximately one year.

Data collection

Client data was collected by OTH during intake and screening and subsequently updated to reflect follow-up and LTC results. De-identified, retrospective data from a two-year screening period were provided by OTH for analysis. Data included screening dates and locations, client demographic information, HIV risk factors, and HIV screening results. Results of an anonymous post-screening satisfaction survey were also provided. The Mass General Brigham Institutional Review Board recommended this study (#2019P003138) as non-human subjects research exempt from continuing review.

Data analysis

Descriptive statistics are presented as frequencies and percentages for categorical variables and as means and standard deviations for continuous variables. Bivariate analyses were used to explore the relationships between client characteristics (race/ethnicity and sex/gender) and the presence of HIV risk factors and previous HIV testing. Pearson’s Chi-Square was used to determine significance, except when the sample size was prohibitively small, in which case Fisher’s exact t-test was used. Statistical significance was defined as p < 0.05. Odds ratios were calculated using open-source software via Vassar College at vassarstats.net.

Few assumptions were made in preparation for data analysis. All data were self-reported, except for HIV screening results. A review of the data indicated several repeat clients. A small minority of clients were either unsure or chose not to report if they had ever been tested for HIV, in which case they were considered to have never been tested. Similarly, very few clients did not respond to questions about race, which were added to the “prefer not to say” category. In all other instances of missing data, the variable was excluded from the analysis. 

Role of funding source

There were no sponsors or funding for this study.

## Results

A total of 846 individuals underwent HIV screening across 72 screening sessions between October 2017 and November 2019 in Wards 4 through 8 of Washington, DC (Table 1). Participants were an average age of 41.7 years, of near equal sex distribution, and from a range of racial backgrounds. Nearly 96% of clients completed HIV screening after registration. Among these, six individuals screened positive for HIV (Table 2). Two were known positives who indicated they were linked to care and adherent to antiretroviral therapy, while four individuals were new preliminary positives. Approximately 13% of clients had never been tested for HIV. Individuals who self-identified as White were less likely to have ever been tested for HIV than were those of other racial backgrounds (OR 0.50, p = 0.04), and cisgender men were less likely to have ever been tested for HIV than were those of other gender identities (OR 0.53, p = 0.0027).

**Table 1:** Demographics, Risk Factors, and Prior HIV Testing MSM: men who have sex with men

Patient Characteristics	Proportion (%, unless otherwise specified)
Age	
Age, mean	41.7 years
Age, median	39 years
Biological Sex, %, Gender Identity	
Male sex, 51.5%	51.5% Cis male
	0.00% Transgender female
Female sex, 48.5%	48.01% Cis female
	0.49% Transgender male
Race	
Black or African American	82.39%
White or Caucasian	6.62%
Native American or Alaskan Native	1.06%
Asian or Pacific Islander	1.54%
Other	6.15%
Prefer not to say	2.25%
Ethnicity	
Hispanic or Latinx	4.37%
Sexuality	
Estimated % of male clients who identify as MSM	13%
Previous HIV Testing	
Yes	83.69%
No	13.59%
Unsure	1.65%
Prefer not to say	1.06%
Previous HIV Test Result	
Negative	81.21%
Positive	0.71%
Unsure	3.66%
Not applicable	13.48%
Prefer not to say	0.95%
Major Risk Factors for HIV	
At least 1 major risk factor	12.88%
At least 2 major risk factors	3.78%
IV drug use	3.48%
Sharing of needles (among IV drug users)	13.79%
A partner who is HIV+	2.96%
Piercing with an unsterilized needle	3.31%
Engage in sex work	5.08%
History of hepatitis B or C	4.14%

**Table 2:** HIV Screening and PrEP Results with One Tent Health PrEP: pre-exposure prophylaxis

Screening result	% of n = 846
Reactive (preliminary positive)	0.71%
Non-reactive (presumed negative)	99.29%
Did not undergo screening after registration	4.19%
New positives	0.47%
PrEP Education and Linkage	% of n = 644
Heard of PrEP	29.51%
Currently taking PrEP	0.88%
Requested linkage to a PrEP provider	29.19%
Requested same-day PrEP prescription	27.36%

In addition to previous HIV testing, clients were assessed for HIV risk factors and counseled on risk-reduction strategies (Table 3). HIV risk factors included injection drug use with or without needle-sharing, HIV-positive sexual partner(s), body piercing with unsterilized needles, commercial sex acts (sex in exchange for money, drugs, or anything of value), and history of hepatitis B or C. Nearly 13% of clients reported at least one of these risk factors and the prevalence of individual risk factors ranged from approximately 3% to 5%, the most common of which was an engagement in commercial sex acts. Clients with HIV risk factors were no more or less likely to have ever been screened for HIV than those without risk factors (OR 1.89, 95% CI 0.93 - 3.86). Approximately 6.8% of all clients were men who have sex with men (MSM) (13% of all cisgender male clients); as limited data was available on sexual partners, sexual orientation was not considered a risk factor here. Similarly, although 61% of clients reported ever engaging in condomless sex, this was not considered a risk factor due to limited information regarding partners, relationships, and instances of condomless sex. Individuals who self-identified as Black or African American were less likely than individuals of other racial backgrounds to have risk factors for HIV (OR 0.57, p = 0.018), while individuals who self-identified as White were more likely to have risk factors for HIV (OR 2.19, p = 0.0170). With regards to sex and gender, which were self-reported separately, cisgender men were more likely to report risk factors for HIV (OR 1.57, p = 0.029), while cisgender women were less likely to report risk factors for HIV (OR 0.53, p = 0.0028).

**Table 3:** Odds of Risk Factors for HIV or Prior Testing by Race, Ethnicity, and Gender, as Compared to all Other Races, Ethnicities, or Genders N/A: not available

Race, Ethnicity, or Gender	Odds Ratio (CI), P-Value
Race and Ethnicity	Odds of having risk factors for HIV
Black or African American	0.57 (0.36 - 0.91), p = 0.0178
White	2.19 (1.13 - 4.21), p = 0.0170
Native American or Alaska Native	0.00 (N/A), p = 0.3849
Asian or Pacific Islander	0.56 (0.07 - 4.35), p = 0.7106
Hispanic or Latinx	0.81 (0.24 - 2.72), p = 1.0000
Sex and Gender	Odds of having risk factors for HIV
Male	1.57 (1.04 - 2.37), p = 0.0296
Female	0.53 (0.35 - 0.81), p = 0.0028
Transgender male	∞ (N/A), p = 0.1288
Race and Ethnicity	Odds of ever receiving prior HIV test
Black or African American	1.07 (0.63 - 1.80), p = 0.8065
White	0.50 (0.25 - 0.98), p = 0.0409
Native American or Alaska Native	1.27 (0.16 - 10.24), p = 1.0000
Asian or Pacific Islander	0.31 (0.09 - 1.04), p = 0.0680
Hispanic or Latinx	1.33 (0.40 - 4.48), p = 0.7863
Sex and Gender	Odds of ever receiving a prior HIV test
Male	0.53 (0.35 - 0.81), p = 0.0027
Female	1.67 (1.10 - 2.51), p = 0.0146
Transgender male	∞ (N/A), p = 1.0000

PrEP education occurred during the intake of 644 clients (Table 2). Over 70% of clients had never heard of PrEP, less than 1% were using PrEP, and approximately 27% - 29% of clients requested either PrEP prescription or linkage to another PrEP provider. 

Of the 169 clients who participated in the post-screening survey, 95.3% reported they were “very pleased” with their experience and 95.8% reported the process took 15 minutes or less. None rated their experience at less than 4 out of 5 on a Likert scale.

## Discussion

Community-based HIV screening offers access to care for individuals who may typically be missed by the healthcare system and allows providers to meet patients where they are, regardless of time, geography, health literacy, or financial barriers. Others have found success around the world with mobile HIV screening in a variety of settings [13, 15-16]. Herein, we describe a unique pop-up screening program, designed to provide rapid, free HIV screening and PrEP education in high-risk areas while minimizing both barriers to access and associated stigma. 

Population reached

This screening program was well-received by the community, with 846 clients undergoing HIV screening and PrEP education, and post-screening survey results indicating 95.3% were “very pleased” with their experience. Clients screened for HIV represented approximately 96% of those who completed intake, further suggesting the feasibility of the model and acceptability among clients. Nearly 13% of clients had at least one major risk factor for HIV, and another 13% had never been screened for HIV. Neither identification as MSM (13%) nor report of condomless sex (61%) was considered risk factors due to the limited data on partners, relationships, or instances of condomless sex. 

The data suggest that pop-up screening reached a high-risk population. HIV surveillance indicated that the highest risk age groups were 35 - 44 and 45 - 55 years of age [4], a range that comprised approximately 40% of the OTH clients. The racial group historically felt to be at the highest risk, Black or African-American [4, 8], was well-represented, accounting for nearly 83% of OTH clients despite census data indicating 46% of DC residents are Black or African American [18]. Additionally, 13% of clients had never been tested, further suggesting that this model reaches high-risk individuals who may have otherwise not received screening. Individuals who identify as White were less likely to have ever been tested for HIV than were those of other racial backgrounds (Table 3). This variance in HIV testing may be due to either self-perceived risk of HIV and the need for testing or may reflect testing initiatives that target groups typically considered to be at higher risk.

A surprising finding from these data was the association between race and HIV risk factors. While Black or African American individuals are among the highest risk groups for new HIV infection in Washington, DC [4-5], they were less likely to report risk factors than clients of other racial backgrounds. We found that Whites were more likely to report risk factors (Table 3). These findings further emphasize the role of social inequities and structural forces that drive the HIV epidemic. There are many factors, such as residential segregation, social capital, stigma, and psychological influences, that intertwine to explain why Black or African Americans are disproportionately affected by this epidemic [6, 19]. As supported by our findings, lifestyle choices alone do not explain racial inequalities in HIV disease burden, and thus, may be insufficient to appropriately gauge a patient’s true HIV risk.

Efficacy and cost

This program found that 0.47% of 846 clients screened newly positive for HIV. With an estimated 51.1 new cases per 100,000 DC residents each year (0.05%), and many of the approximately 2% - 3% of DC residents living with HIV already linked to care, we believe this rate of new positive screens supports the utility of the program [2, 5]. To further contextualize this data, OTH found a new positive rate of 227 cases per 100,000 residents per year, more than four times higher than the new positive rate found by traditional testing methods in DC, suggesting value added to a healthcare system that seeks to identify undiagnosed, difficult-to-reach cases. Of the six clients who screened positive, two were successfully reached for a 12-month follow-up; one reported successful initial LTC but had since not been retained in care, while the other client was retained in care. The small number of clients who screened positive prohibits further meaningful statistical analysis. The 846 screens and 644 PrEP education sessions were achieved at an estimated operational cost of $138,367 over the 25 months of the study period. While a cost-effectiveness analysis is beyond the scope of this work, non-market benefits (e.g., community engagement, the potential for stigma and risk reduction, PrEP education and referral, and knowledge of HIV status) confer added value to this program.

PrEP education and linkage

Although PrEP is a mainstay of HIV prevention [20], there are significant concerns regarding the need for novel methods of PrEP delivery [21-23], and healthcare providers’ limited knowledge of PrEP may be a contributor to reduced uptake [24]. Accordingly, less than 30% of our clients had ever heard of PrEP, and only 0.88% were taking PrEP. After a brief education, 27.36% of the clients requested a same-day PrEP prescription, though a smaller proportion indicated risk factors suggesting an indication for a prescription. The observed low use of PrEP, despite risk factors, correlates well with the extant literature, and this pop-up model may be well-designed to reduce the gap both in education and prescription. Further data regarding PrEP uptake and long-term adherence from this program are needed. 

Program feasibility strengths

While community-based screening comes in a variety of formats, brick-and-mortar clinics and mobile units can be limited in efficacy by spatiotemporal requirements, operational costs, and staffing needs. This rapid pop-up model is designed to reduce such barriers as follows:

Spatiotemporal Requirements

Traditional clinics are often subject to limited hours of operation and are not always located in the highest-risk areas of DC. Some clinics only have HIV screening and LTC services at certain hours of the day, while others may have prohibitively long wait times. All such clinics require individuals to know their physical locations and to take the initiative to seek out their services. Individuals unaware of their risk factors or their preventative care needs maybe even less likely to seek care. Regarding mobile vans, these are conceivably difficult to park in public spaces that have the highest foot traffic. 

The pop-up tent model allows for screening on weekends and at multiple locations throughout the city where mobile units might not typically have the space for operations. OTH targets areas, such as grocery stores, pharmacies, and laundromats, where visibility reduces the burden on individuals to seek care. 

Operational Costs

OTH spent $138,367 on operations over 25 months of screening or approximately $66,416 per year, a significantly lower cost of operation than a typical brick-and-mortar clinic or even a mobile van screening program [16]. The use of a tent, which costs approximately $500, is a major factor in the cost reduction. Screening kits were provided free of charge by the DC Department of Health and thus do not factor into the operational cost. Expenses such as insurance, licensing, equipment, and others are included in operations costs.

Staffing

Staffing models influence both feasibility and operational costs. The screening kit used by OTH is under CLIA-waived status, and as the operational focus is specifically HIV screening and PrEP education, clinicians are not required on-site. PrEP prescription and other medical care are instead provided by clinical partners during referral or LTC. Screening sessions are thus performed by volunteers who are trained with knowledge and skills to provide rapid HIV screening and PrEP education, risk factor assessment, and LTC. These non-clinical volunteers can also enhance their engagement by serving in leadership and education roles. As community engagement is considered a critical component of improving outcomes for PLWH [25], this unique model contributes to community engagement around HIV and provides an opportunity for young adults to engage in preventative HIV services. 

Reducing Stigma

It is difficult to measure how an HIV screening program might directly reduce the stigma surrounding HIV and HIV screening. Fear and misunderstanding about transmission may be a driver of stigma, perhaps a relic of early misconceptions of HIV. For this reason, OTH opted to make education about HIV and PrEP a centerpiece of the program, a decision that may help reduce bias and misconception. The visibility of the program, in open spaces available to the public as opposed to behind closed doors of clinics, may also contribute to normalizing HIV screening and other preventative health measures. A small minority of persons who underwent intake ultimately chose not to receive HIV screening (n = 37, 4.19%). While no data are available regarding their decision to forego screening, a variety of factors, such as wait time or anxiety about results, could act as deterrents.

Limitations

Several limitations are worth noting. LTC and lost to follow-up (LTFU) are established challenges of many organizations, with as much as one-third LTFU at 12 months and more than half not retained in care [26]. Many factors likely contribute to LTFU, including limited transportation, time restrictions, insurance needs, anxiety regarding HIV diagnosis, relocation, and dissatisfaction with a provider. While the timely receipt of screening results (an area where the OTH model excels) is associated with LTC [27], there is significant room for improvement in successful LTC. The benchmark for this metric does not appear to be established, and there was a small total number of OTH clients who screened positive. However, replication of this model may benefit from incorporating additional LTC resources, such as patient navigators or case managers. Additionally, while limited on-site staffing and other resources may reduce throughput time and increase client satisfaction, this is coupled with the drawback of limited opportunities to address other medical concerns, provide on-site confirmatory testing, or initiate treatment. Furthermore, while the INSTI® HIV 1/2 Rapid Antibody Kit is reported to be very accurate [28], there is limited data on the final confirmatory testing of OTH clients who screened positive. Finally, while the pop-up tent model has the benefit of markedly reduced operation costs, data are not available for a cost-effectiveness analysis at this time.

## Conclusions

The OTH pop-up model is a feasible and effective way of increasing community-based HIV screening and identifying a higher rate of newly positive HIV cases than traditional testing methods in the District of Columbia. By reducing access barriers, stigma, and costs, the OTH model provides a unique and needed platform for improving community engagement, HIV education, risk reduction strategies, and knowledge and prescription of PrEP. Organizations with a wealth of resources and staff may benefit from the judicious application of those resources to their community-based screening, as these may decrease efficiency and patient satisfaction. However, after the conclusion of the pilot program reported here, this model was later adapted to include a COVID-19 screening program, alongside HIV screening, which did require an increased number of staff members. LTC remains a challenge in pop-up HIV screening, but organizations should maintain a clear LTC plan and same-day LTC should be prioritized. Patient navigators and enhanced patient contact should also be considered, as these improve retention in care in a variety of settings, and may also be beneficial when implementing a pop-up model. We find that this healthcare delivery model reaches critical populations with risk factors for HIV and can be easily replicated to provide other health maintenance activities, which may be important during both the COVID-19 pandemic and the ongoing global HIV epidemic.
